# Investigating the impaired sensorimotor gating in adult mice following foetal exposure to inflammatory mediators

**DOI:** 10.1186/1753-6561-6-S4-P49

**Published:** 2012-07-09

**Authors:** M Tsakok, H Stolp, Z Molnár

**Affiliations:** 1 University of Oxford, UK; 2 Department of Physiology, Anatomy and Genetics, University of Oxford, UK

## Introduction

Maternal infection during pregnancy is a risk factor for certain psychiatric illnesses with a neurodevelopmental component, such as schizophrenia and cerebral palsy. Pathology is thought to result from the exposure of the foetal brain to pro-inflammatory cytokines stimulated during the inflammatory response that influence developmental processes via their actions on neurones and glial cells. In experimental animals, behavioural outcomes relevant to such disorders have been observed in the offspring of infected dams - this study therefore sought to determine whether behavioural change was present in our murine model of immune activation.

## Methods

The animal model involved lipopolysaccharide induction of maternal inflammation in pregnant female mice. Sensorimotor gating was assessed using the acoustic startle response - the involuntary contraction of the limbs following an unanticipated, loud, acoustic stimulus. If a weaker pre-stimulus is applied prior to this stimulus, the startle response is attenuated, and this is termed prepulse inhibition. The independent variable was the magnitude of the pre-pulse stimulus, whilst the dependent variable was the percentage of prepulse inhibition. Separate one-way analyses of variance (ANOVAs) were conducted to analyse the effects of treatment and gender on pre-pulse inhibition and post-hoc Bonferroni correction for multiple comparisons was performed with p<0.05 considered significant. Another behavioural test, burrowing activity, was also investigated as a sensitive measure of general behavioural abnormality.

## Results

Maternal inflammation induced by lipopolysaccharide (LPS) on gestation day 18 caused significant impaired sensorimotor gating in the adult offspring that is most clearly seen when the animals are subjected to a prepulse of +16dB. However, maternal immune activation had no effect on burrowing activity - a more general aspect of rodent behaviour.

## Conclusion

We have made the significant finding of a long-term behavioural change in a mouse model in which very clear deficits in sensorimotor gating were observed. In addition, we suggest that general behaviour is not affected by maternal inflammation. Our model of maternal inflammation corroborates the findings of other groups in a similar field. This work thus paves the way for investigating the long unanswered question of which cellular and molecular mechanisms are involved in the developing brain's response to inflammation.

